# Aluminum Nitride Transition Layer for Power Electronics Applications Grown by Plasma-Enhanced Atomic Layer Deposition

**DOI:** 10.3390/ma12030406

**Published:** 2019-01-28

**Authors:** Heli Seppänen, Iurii Kim, Jarkko Etula, Evgeniy Ubyivovk, Alexei Bouravleuv, Harri Lipsanen

**Affiliations:** 1 Department of Electronics and Nanoengineering, Aalto University School of Electrical Engineering, P.O. Box 13500, FI-00076 Aalto, Finland; iurii.kim@aalto.fi (I.K.); bour@mail.ioffe.ru (A.B.); harri.lipsanen@aalto.fi (H.L.); 2 St. Petersburg Academic State University, Ul. Khlopina 8/3, 194021 Saint Petersburg, Russia; 3 Department of Chemistry and Materials Science, Aalto University School of Chemical Engineering, P.O. Box 16100, FI-00076 Aalto, Finland; jarkko.etula@aalto.fi; 4 ITMO University, Kronverksky pr. 49, 197101 Saint Petersburg, Russia; ubyivovk@gmail.com

**Keywords:** AlN, ALA, ALD, buffer layers, transition layer, MOCVD, regrowth

## Abstract

Aluminum nitride (AlN) films have been grown using novel technological approaches based on plasma-enhanced atomic layer deposition (PEALD) and in situ atomic layer annealing (ALA). The growth of AlN layers was carried out on Si<100> and Si<111> substrates at low growth temperature. The investigation of crystalline quality of samples demonstrated that PEALD grown layers were polycrystalline, but ALA treatment improved their crystallinity. A thick polycrystalline AlN layer was successfully regrown by metal-organic chemical vapor deposition (MOCVD) on an AlN PEALD template. It opens up the new possibilities for the formation of nucleation layers with improved quality for subsequent growth of semiconductor nitride compounds.

## 1. Introduction

III-nitrides including aluminum nitride (AlN) are attractive materials for high-power electronics, especially for high electron mobility transistors (HEMTs). The wide band-gap, high critical electric field, and high thermal conductivity of AlN provides an opportunity for HEMTs to withstand demands of aggressive environments [1]. Expensive bulk gallium nitride (GaN) or AlN substrates enhance interest to growth of wide band-gap semiconductors on low-cost silicon (Si), sapphire (Al${}_{2}$O${}_{3}$) or silicon carbide (SiC) substrates [2,3,4,5]. Conventionally, III-N layers can be grown on Si substrates by metal-organic chemical vapor deposition (MOCVD) [6] or molecular-beam epitaxy (MBE). However, the high temperatures required for epitaxial growth, mismatches in the lattice and thermal expansion coefficient can result in the formation of numerous dislocations or even cracks in the GaN film on account of large tensile stress either during growth or during post-growth cooling [7,8]. The growth of AlN transition layer (TL) following by related buffer Al${}_{x}$Ga${}_{1-x}$N layers are used between Si and GaN to overcome these issues [9,10,11]. Although the use of different step graded AlGaN and AlN buffer layers have been widely studied [12,13,14,15,16,17], the nucleation of the AlN layer on Si substrate is not well understood [18]. Moreover, the thick buffer layer is required to bury dislocations [19], which leads to an increase in the thickness of the device and inefficient consumption of materials.

One of the possible alternatives for thick buffer layers is use of thin AlN layer grown with plasma-enhanced atomic layer deposition (PEALD) [20]. Although it is commonly believed that atomic layer deposition (ALD) produces amorphous AlN film [21], the recently introduced in situ atomic layer annealing (ALA) [22] results in high-quality, hypothetically single-crystalline, AlN growth. Therefore, PEALD enables fabrication of low-cost templates with high quality AlN transition layer for following epitaxial steps of the device fabrication process. In the in situ ALA method, an extra plasma pulse with the plasma gas only is applied on the surface after each cycle, which provides extra energy for the bond formation and therefore enhances the crystallization process. Nevertheless, the properties of such ALA grown AlN films on different substrates have not been extensively studied previously. In the present work, the effect of Si<100>, Si<111> substrates, as well as plasma treatment time and power on film growth are investigated.

## 2. Results and Discussion

**Figure 1:**
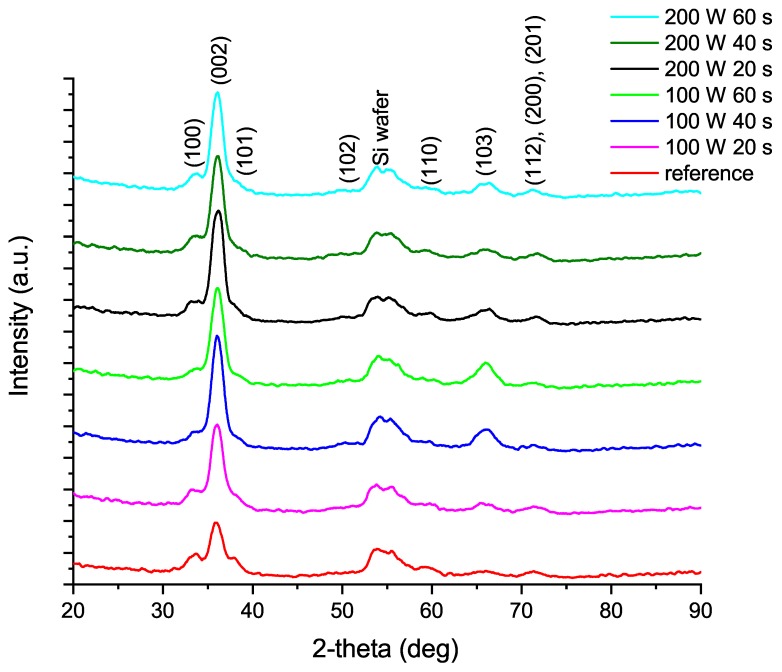
The 2-theta GIXRD patterns of the ALN layers on Si<100>. Reference sample was grown without ALA treatment. The results are presented in the same order as in the legend.

The effect of the plasma treatment time and power on the film crystallization is shown in the grazing incidence X-ray diffraction (GIXRD) patterns in Figure 1. The increase of the intensity from (002) peak has previously been reported in literature [21,22,23,24] and indicates a hexagonal wurtzite aluminum nitride layer. Recently, Shih et al. [22] showed that the effect of ALA treatment results in epitaxial AlN film on sapphire. Figure 1 on the other hand shows also other orientations growing on <100> silicon substrate. This can be explained by the differences between sapphire and silicon substrates. The hexagonal lattice of sapphire enables fully epitaxial growth of AlN in contrast with the cubic lattice of the silicon substrate.

The effect of the ALA treatment is already visible with only 100 W plasma power and 20 s treatment time. Compared to the reference sample without in situ ALA treatment (red line in Figure 1), the intensity increase of the (002) AlN peak is clear in all samples of the series. ALA treatment with 200 W plasma power for 60 s should provide sufficient energy to form the most epitaxial film due to surface heating [22]. Nevertheless, 200 W ALA treatment for 20 s already shows a comparably pronounced (002) peak. To decrease process times and costs, a shorter process of comparable result is often preferable. GIXRD provides a sensitive and facile measurement of thin film crystallinity without an interfering substrate background, but is less comprehensive in studying fully epitaxial films when compared to reciprocal space mapping.

The preference of (002) growth can be explained by the required energy to form Al and N bonds. The (002) plane in wurtzite lattice is formed by two bonds with different lengths. The longer bond requires lower energy and forms only the plane (100) or has no preferred orientation. The shorter bond requires higher energy to form. The planes (002) and (101) are formed only when both of the bond types exist in a well-formed order [25]. The formation of wurtzite lattice during deposition of AlN is explained in closer detail by Xu et al. [25] and Zhang et al. [26]. The formation of (100) plane can also be noticed when comparing the (100) peak from the reference sample to any of the ALA treated samples; the intensity of the (100) peak remains constant regardless of the applied plasma power or time. This demonstrates the substrate imitating nature of epitaxial ALD growth as the it takes place at the available surface sites and therefore automatically copies the surface structure. Subsequently the sufficient thermal energy from the plasma forms the preferred orientation of AlN. This effect is most evident when comparing the 100 W 20 s and 40 s ALA treated samples with the reference; the intensity of (100) remains constant as the intensity of (002) grows.

**Figure 2:**
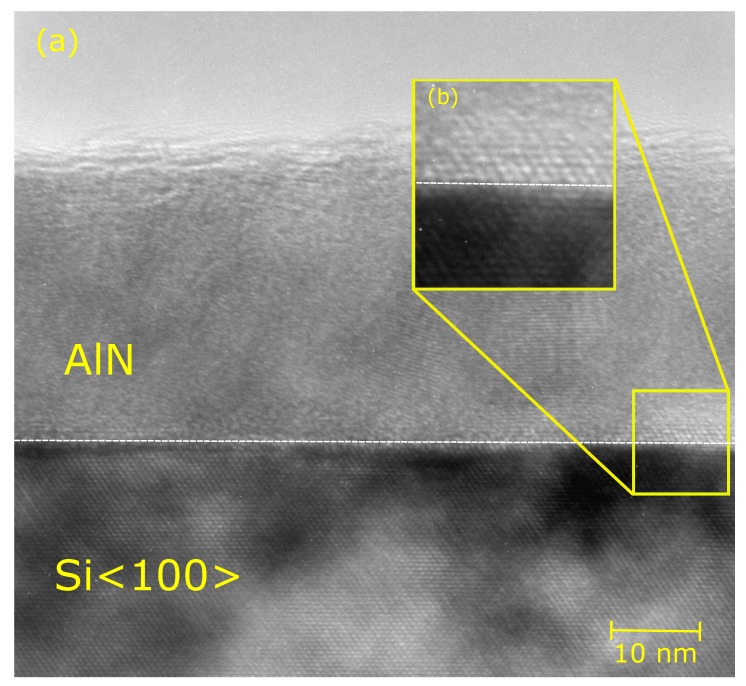
(**a**) TEM cross-section image of 30 nm AlN grown on Si<100> with a in situ ALA treatment at plasma power of 200 W and 60 s; (**b**) Enlarged interface of the Si<100> substrate and AlN of the cross-section.

The 200 W and 60 s sample was further investigated with TEM. Figure 2 shows the TEM cross-section image of the interface between Si<100> and ALA treated AlN, where a thin about 3 nm epitaxial AlN layer is distinct at the interface. This epitaxial layer at the interface is further noticed from the GIXRD patterns from the corresponding (100) peak apparent in all of the measured samples. According to the TEM image, the microstructure of the film is polycrystalline. This is in agreement with the GIXRD results in Figure 1, and further suggests that shorter ALA process times of 20 s can be preferable. The thickness of the film is in accordance with the approximately 28 nm thickness obtained from both ellipsometry and X-ray reflectometry (XRR) shown in the Supplementary Information (Figure S1). Identical substrates with silicon dioxide layer on top of the bulk silicon were used for the matching of XRR data. The results from the matching are presented in Table 1.

**Figure 3:**
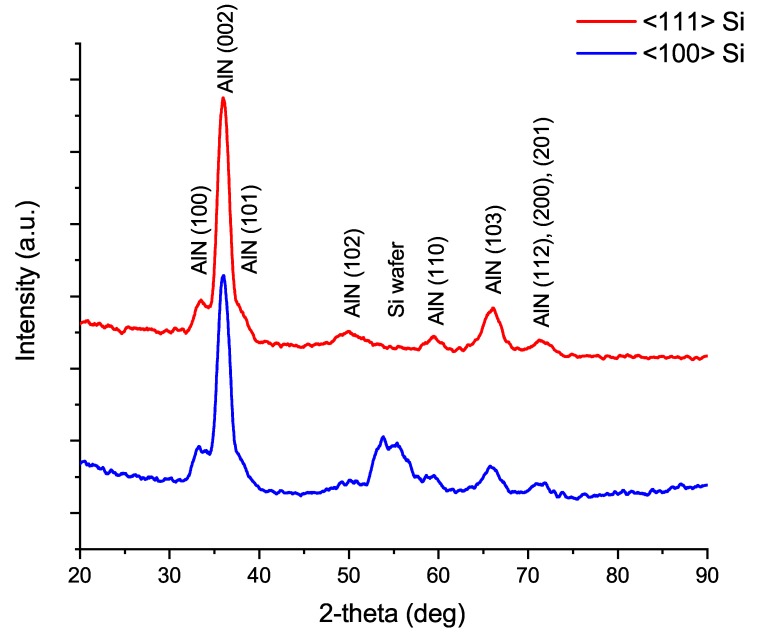
The 2-theta GIXRD patterns of 200 W and 20 s in situ ALA treated layers on Si<111> (**top**) and Si<100> (**bottom**).

The density from the XRR matching was 3.0 g/cm${}^{3}$ which is quite close to the theoretical density 3.26 g/cm${}^{3}$ of AlN [27]. The top layers of AlN seem less dense and are probably caused by surface oxidation in contact with air. This, as well as any alignment errors can affect the matching of the XRR data to simulation and hence density [28]. The growth of the ALA treated AlN was also investigated on less mismatched Si<111> substrate. The GIXRD patterns from the films are presented in Figure 3. Since the (002) peak is clearly pronounced in all of the samples in the plasma power and time series, the process with 200 W plasma power and 20 s ALA treatment was chosen for further investigation with other silicon substrates due to the fastest processing time and clear difference in crystallinity to the twin process with 100 W plasma power.

The effect of preferred growth of (002) plane is apparent on every substrate. Previous research [26] found the lattice of Si<111> to positively affect on the hexagonal AlN formation. The effect was also detected in this research as a higher intensity (002) peak from the Si<111> substrate compared to the peak from Si<100>. The (100) peak, however, remains as high in both of the bare silicon substrates.

**Figure 4:**
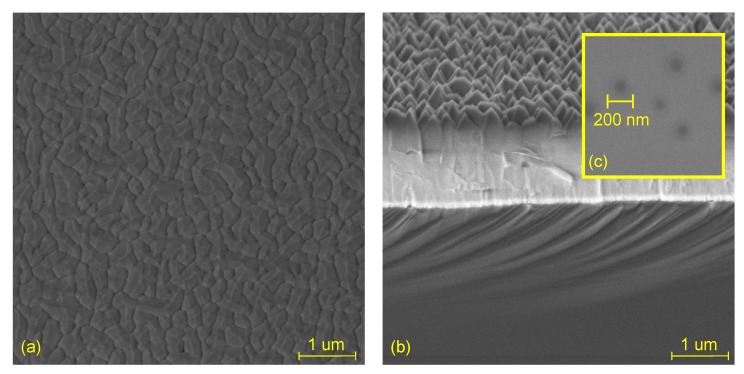
Top (**a**) and cross-sectional (**b**) SEM images of regrown AlN layer by MOCVD on top of ALD AlN transition layer (TL); (**c**) Top SEM image of blisters in the TL AlN grown on Si<100> with an in situ ALA treatment at plasma power of 100 W and 40 s.

The proposed ALD AlN layer is suggested to act as the transition layer (TL) between substrate and further III-N layers e.g., in high-power applications. To demonstrate the general feasibility of ALA treatment method, we fabricated a sample with thick (∼2 μm) AlN layer presented in Figure 4.

The 100 W and 40 s sample was used as a template for AlN MOCVD regrowth. Figure 4a shows a top scanning electron microscope (SEM) view of regrown AlN layer by MOCVD. The interfaces between AlN grains (dark strips), which are clearly seen in the image, allow us to deduce that the film has a columnar structure with an average diameter of 100 nm. The cross-sectional view presented in Figure 4b also reveals high roughness of the top layer. The high roughness is probably caused by the polycrystalline ALD film. Regrowth of MOCVD AlN film on top of the ALD AlN film was successful despite so-called blisters on the ALD layer surface presented in Figure 4c. They are probably caused by H${}_{2}$ vapor traps [29] due to insufficient purge times or poor flow geometry of the chamber. The *θ-2θ* XRD pattern of the regrown AlN by MOCVD on top of PEALD template is shown in the Supplementary Information (Figure S2). The peaks at (002), (101) and (004) define that AlN layer was polycrystallized. However, the surface quality and AlN crystallographic orientation of grown MOCVD layer should be further analyzed. Nevertheless, already this possibility for the regrowth clearly indicates the ALD AlN transition layer as a potential replacement for current thick buffer layers in III-N devices.

## 3. Conclusions

Highly (002) oriented AlN transition layer grown in PEALD with in situ ALA treatment was demonstrated. The effect of the plasma power and duration in the in situ ALA treatment was studied in more detail on Si<100>. Si<111> was found to be the most promising of the silicon substrates, since the effect of the crystallization in the wanted (002) direction was distinct. This, however, should be further verified with reciprocal space mapping of the films. The effect of different ALA treatments crystallization preferring (002) direction on Si<111> remains for future studies. In addition, we demonstrated the use of AlN layer grown in PEALD as a transition layer for further MOCVD regrowth, indicating that PEALD with ALA treatment provides a viable pathway to fabricate III-N devices on silicon substrates.

## 4. Materials and Methods

Aluminum nitride was grown in a plasma-enhanced ALD reactor (TFS-500, Beneq Oy, Espoo, Finland). Trimethylaluminum (TMA) and ammonia (NH${}_{3}$) were used as precursors and argon (Ar) as the carrier and plasma gas. The depositions were made at 300 ${}^{{}^{\circ}}$C based on the process presented by Bosund et al. [21] with 0.3 s and 3 s TMA pulse and purge times and 15 s and 5 s NH${}_{3}$ pulse and purge times. The in situ ALA was varied from the reference 0 s to 20 s, 40 s and 60 s with plasma powers 100 W and 200 W. ALA was performed directly after each ALD cycle with only Ar as plasma gas. The plasma power for the reference sample was 200 W for the NH${}_{3}$ pulse. The samples were cooled down in nitrogen (N${}_{2}$) atmosphere after the deposition. The deposited thickness was around 28 nm and was verified with an ellipsometer (Plasmos SD2300, Philips Analytical Technology GmbH, Munich, Germany) right after the cool down. The crystallinity, thickness, density and roughness of the films were determined with XRR and GIXRD measurements using a Rigaku SmartLab diffractometer. Surface optimized grazing incidence XRD scans were measured in parallel beam mode at an incidence angle of 0.4 ${}^{{}^{\circ}}$ using a 9 kW rotating anode with a germanium monochromator (Cu Kα${}_{1}$ radiation) and a 2D detector (HyPix-3000).

## Figures and Tables

**Table 1:** Thickness, density and roughness from matching of XRR data of the ALA treated films on Si<100> substrate.

Sample	Thickness (nm)	Density (g/cm${}^{3}$)	Roughness (nm)
100 W 20 s	28.3	3.0	1.5
100 W 40 s	28.3	3.0	1.5
100 W 60 s	26.6	3.0	1.3
200 W 20 s	28.8	3.1	1.5
200 W 40 s	28.6	3.1	1.6
200 W 60 s	27.8	3.0	1.7

## Supplementary Materials

The following are available online at http://www.mdpi.com/1996-1944/12/3/406/s1, Figure S1: The measured XRR (red) and fitted (blue) curves from the plasma series and the reference sample, Figure S2: *θ*-2*θ* XRD scan of the regrown MOCVD AlN.
