# Supplementary material for: Aluminum Nitride Transition Layer for Power Electronics Applications Grown by Plasma-Enhanced Atomic Layer Deposition
Source: Materials (Basel). 2019 Jan 28;12(3):406. doi: 10.3390/ma12030406 (PMC6384632; doi:10.3390/ma12030406)
Supplement: Supplementary file 1 [file materials-12-00406-s001.pdf]

materials

MDPI

Article

Aluminum Nitride Transition Layer for Power Electronics Applications Grown by Plasma-Enhanced Atomic Layer Deposition

Heli Seppänen [1,*] , Iurii Kim [1,2] , Jarkko Etula [3] and Evgeniy Ubyivovk [4], Alexei Bouravleuv [1,2] and Harri Lipsanen [1]

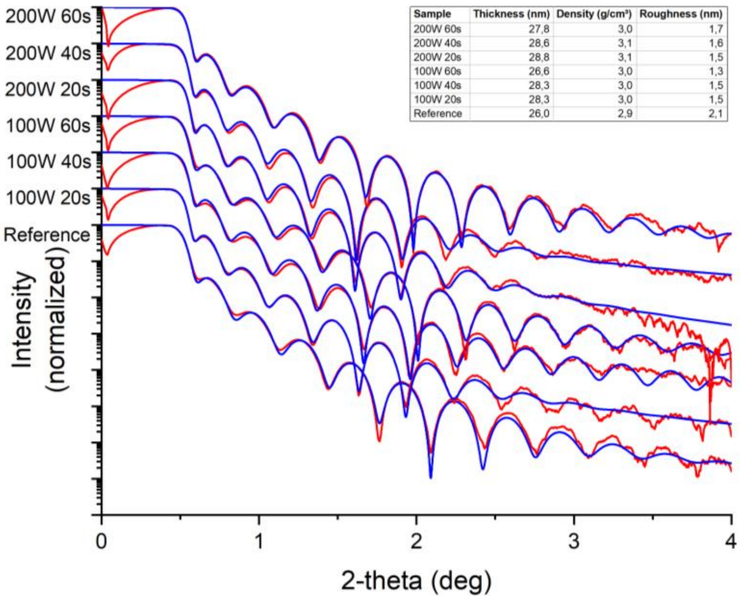

Figure S1. The thickness of the film is in accordance with the approximately 28nm thickness obtained from both ellipsometry and X-ray reflectometry.

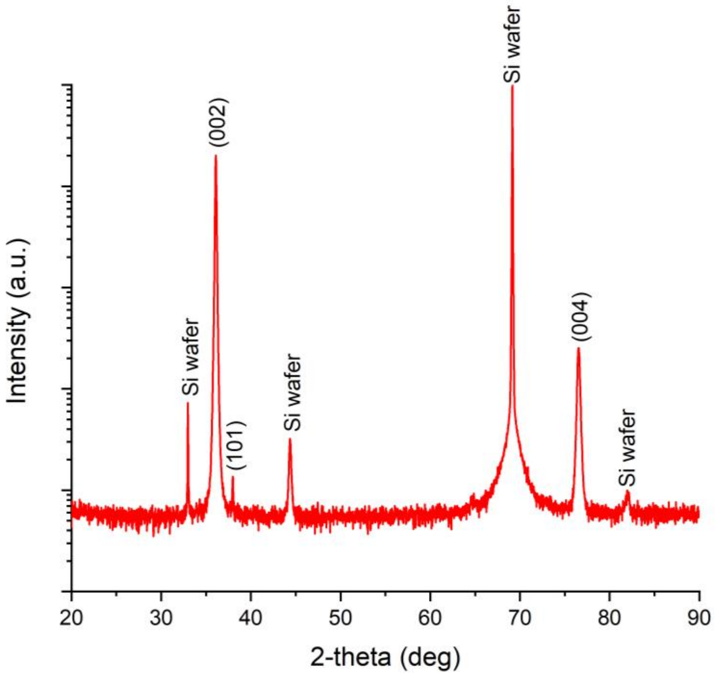

Figure S2. The θ-2θ XRD pattern of the regrown AlN by MOCVD on top of PEALD template.
